# A Nomogram Incorporating Neutrophil-to-Lymphocyte Ratio and Squamous Cell Carcinoma Antigen Predicts the Prognosis of Oral Cancers

**DOI:** 10.3390/cancers15092492

**Published:** 2023-04-26

**Authors:** Yao-Te Tsai, Chia-Hsuan Lai, Geng-He Chang, Cheng-Ming Hsu, Ming-Shao Tsai, Chun-Ta Liao, Chung-Jan Kang, Yuan-Hsiung Tsai, Yi-Chan Lee, Ethan I. Huang, Ming-Hsien Tsai, Ku-Hao Fang

**Affiliations:** 1 Department of Otorhinolaryngology-Head and Neck Surgery, Chang Gung Memorial Hospital, Chiayi 60040, Taiwan; yaote1215@gmail.com (Y.-T.T.); genghechang@gmail.com (G.-H.C.); scm00031@gmail.com (C.-M.H.); b87401061@gmail.com (M.-S.T.); ehuang.mdphd@gmail.com (E.I.H.); 2 College of Medicine, Chang Gung University, Taoyuan 330036, Taiwan; chiahsuan7092@gmail.com (C.-H.L.); liaoct@cgmh.org.tw (C.-T.L.); handneck@gmail.com (C.-J.K.); russell.tsai@gmail.com (Y.-H.T.); alberttylee@gmail.com (Y.-C.L.); b9302094@cgmh.org.tw (M.-H.T.); 3 Department of Radiation Oncology, Chang Gung Memorial Hospital, Chiayi 60040, Taiwan; 4 Department of Otorhinolaryngology-Head and Neck Surgery, Chang Gung Memorial Hospital, Taoyuan 333423, Taiwan; 5 Department of Diagnostic Radiology, Chang Gung Memorial Hospital, Chiayi 60040, Taiwan; 6 Department of Otorhinolaryngology-Head and Neck Surgery, Chang Gung Memorial Hospital, Keelung 20401, Taiwan; 7 Department of Otorhinolaryngology-Head and Neck Surgery, Chang Gung Memorial Hospital, Kaohsiung 833253, Taiwan

**Keywords:** oral cavity cancer, squamous cell carcinoma inflammatory index, nomogram, biomarker

## Abstract

**Simple Summary:**

We introduced a novel squamous cell carcinoma inflammatory index (SCI) derived by multiplying the serum squamous cell carcinoma antigen and neutrophil-to-lymphocyte ratio values for individuals with operable oral cavity squamous cell carcinomas (OSCCs). The prognostic value of SCI was explored by retrospectively analyzing data from 288 patients with a diagnosis of primary OSCC between January 2008 and December 2017. The current results demonstrated that patients with a high SCI (≥3.45) were associated with worse disease-free survival and overall survival than those with a low SCI (<3.45). An elevated preoperative SCI (≥3.45) predicted adverse overall survival (hazard ratio [HR] = 2.378; *p* < 0.002) and disease-free survival (HR = 2.219; *p* < 0.001) in a multivariable analysis. The constructed nomogram enables the clinical utility of the SCI and provides accurate OS predictions. Our findings indicate that SCI is a valuable and promising biomarker that is highly associated with patient survival outcomes in OSCC.

**Abstract:**

We introduced a novel squamous cell carcinoma inflammatory index (SCI) and explored its prognostic utility for individuals with operable oral cavity squamous cell carcinomas (OSCCs). We retrospectively analyzed data from 288 patients who were given a diagnosis of primary OSCC from January 2008 to December 2017. The SCI value was derived by multiplying the serum squamous cell carcinoma antigen and neutrophil-to-lymphocyte ratio values. We appraised the associations of the SCI with survival outcomes by performing Cox proportional hazards and Kaplan–Meier analyses. We constructed a nomogram for survival predictions by incorporating independent prognostic factors in a multivariable analysis. By executing a receiver operating characteristic curve analysis, we identified the SCI cutoff to be 3.45, and 188 and 100 patients had SCI values of <3.45 and ≥3.45, respectively. The patients with a high SCI (≥3.45) were associated with worse disease-free survival and overall survival than those with a low SCI (<3.45). An elevated preoperative SCI (≥3.45) predicted adverse overall survival (hazard ratio [HR] = 2.378; *p* < 0.002) and disease-free survival (HR = 2.219; *p* < 0.001). The SCI-based nomogram accurately predicted overall survival (concordance index: 0.779). Our findings indicate that SCI is a valuable biomarker that is highly associated with patient survival outcomes in OSCC.

## 1. Introduction

Oral cavity cancer has the fourth-highest incidence in men in Taiwan [1]. Oral cavity squamous cell carcinoma (OSCC) was revealed to constitute over 90% of the cases of oral cavity cancer, which can typically be treated with radical resection with or without adjuvant therapy [2]. Despite advances in the treatment of OSCC, disease recurrence remains a significant problem. Studies have shown that up to 40–60% of patients with OSCC may experience disease recurrence within 5 years of treatment [3]. In addition to the tumor–node–metastasis (TNM) classification, several important prognostic factors for OSCC have been reported, such as lymphovascular invasion (LVI) [4], depth of invasion (DOI) [5], perineural invasion (PNI) [6], and extranodal extension (ENE) [2,7]. However, the aforementioned factors can be identified only after surgery. Accordingly, the identification of preoperative prognostic biomarkers may be helpful in predicting the prognosis of OSCC and selecting appropriate treatment strategies for individual patients.

The tumor-related protein squamous cell carcinoma antigen (SCC-Ag) was first identified in patients with cervical cancer by Kato et al. [8]. SCC-Ag belongs to a superfamily of serine proteinase inhibitors and plays a crucial role in tumorigenesis and cancer metastasis by increasing cell migration and inhibiting apoptosis [9]. Studies have reported SCC-Ag to be involved in the prognosis of numerous human squamous cell cancers, including esophageal [10], bladder [11], anal [12], lung [13], cervical [14], and OSCC [15]. Huang et al. included 142 patients with OSCC in their study and demonstrated that SCC-Ag at a level greater than or equal to 2.0 ng/mL has a significant association with the adverse pathological features and prognosis of OSCC [16]. In addition, cancer-related inflammatory responses have been reported to be closely associated with cancer development and progression [17,18]. Studies have indicated that several inflammatory biomarkers can be used as prognostic indicators for human cancers [19] and that a high neutrophil-to-lymphocyte ratio (NLR) can negatively influence the prognosis of individuals with OSCC [20]. However, the NLR is not a tumor-specific biomarker, which has prevented the development of a rationale supporting its use in cancer management. The results of the previously mentioned studies indicate that the combined evaluation of SCC-Ag and NLR may offer complementary information for OSCC prognostication. Nevertheless, to the best of our knowledge, the potential combined value of SCC-Ag and NLR as a prognostic biomarker has yet to be discussed in the literature. Accordingly, to address this gap, we herein present a novel squamous cell carcinoma inflammatory index (SCI) that we derived by multiplying serum SCC-Ag and NLR values. Given that the SCI may provide a comprehensive assessment of tumor–host interactions, we hypothesized that the SCI would have significant associations with survival outcomes and explored the SCI’s prognostic utility by retrospectively assessing patients that have been surgically treated for OSCC.

## 2. Materials and Methods

### 2.1. Patients

Our retrospective cohort study involved analyzing medical data derived from 313 consecutive patients who were pathologically determined to have primary OSCC between January 2008 and December 2017 at our hospital. All patients received radical surgery, which was followed by adjuvant therapy if indicated. The patients were excluded if they had a diagnosis of synchronous cancer or a cancer history (*n* = 7), had undergone neoadjuvant treatment before surgery (*n* = 6), had autoimmune diseases or acute infections prior to surgery (*n* = 1), or had missing laboratory or follow-up data (*n* = 11). We determined the data from 288 patients to be suitable for inclusion in the formal analysis. The study methods and procedures complied with the principles of the Declaration of Helsinki. In addition, this executed study protocol was granted approval by the Institutional Review Board of our hospital (number: 202300107B0).

### 2.2. Collection of Study Data

The medical personnel obtained the clinical data of the included patients by reviewing the electronic medical chart system of our hospital. Before the surgery, all patients had been subjected to a comprehensive cancer staging workup in accordance with our institutional guidelines. Clinicopathological features, including sex, age, primary tumor location, pathological cancer stage (American Joint Committee on Cancer Staging Manual, 8th edition, 2018), LVI, ENE status, PNI status, DOI, nearest surgical margin, and tumor differentiation, were extracted and analyzed as prognostic factors. For each patient, the internal tumor conference results and the institutional guidelines were used as the basis to determine whether they required adjuvant therapy within 6 weeks postsurgery [21]. Any underlying comorbidities were defined and quantified using the Charlson comorbidity index (CCI) [22]. The patients were considered to be consumers of alcohol, cigarettes, or areca nuts if they reported having >1 alcoholic beverage every week for >6 months, smoking ≥ 1 pack each day for >1 year, or chewing ≥ 2 areca nuts each day for >1 year, respectively [23].

### 2.3. Measurement of Serum Indices

This study used blood samples obtained one week before surgery. The serum SCC-Ag levels (reference cutoff value: 2.5 ng/mL) were obtained using a chemiluminescent microparticle immunoassay (Abbott Japan Co. Ltd., Tokyo, Japan). We obtained our patients’ blood biochemistry values by using a Cobas 8000 biochemistry analysis system, which is an automated system (Roche Hitachi, Rotkreuz, Switzerland). In addition, we employed a Sysmex SE-9000 hematology analysis system to derive the peripheral blood cell counts (Sysmex, Kobe, Japan). We defined the SCI by using the following equation: SCI = SCC-Ag × NLR.

### 2.4. Follow-Up and Study Endpoints

All patients had been scheduled for regular clinic visits (every 2 and 3 months during the first and second years, respectively, and every 6 months thereafter) and periodic imaging surveillance of the head, neck, and chest. The study endpoints were as follows: disease-free survival (DFS), which was specified as the period spanning from the surgery date to the date of receiving a diagnosis for distant metastasis or locoregional recurrence, death, being censored from the study, or the last follow-up appointment for the alive patients; and overall survival (OS), which was specified as the period spanning from the surgery date to that of all-cause mortality, being censored from the study, or the last follow-up appointment for the alive patients. Telephone interviews and electronic medical records were used to obtain the patients’ follow-up data. We determined our median (range) follow-up period to be 40.1 (3.5–122.4) months, with the last follow-up date being 31 December 2019.

### 2.5. Statistical Analysis

This study presents the categorical variables as percentages and absolute numbers for the study cohort. We determined the normality of the data by performing the Kolmogorov–Smirnov test. This study also presents the continuous variables as medians and interquartile ranges if the data were non-normally distributed. To derive the best serum index cutoff values, we executed a receiver operating characteristic (ROC) curve analysis along with a Youden’s index calculation. In addition, we estimated the areas under the ROC curves (AUCs) for OS predictions in order to compare the prognostic discriminatory ability of the SCI with that of other indices. The study patients were grouped in accordance with the optimal SCI cutoffs, and we compared the clinicopathological variables of the patients with low and high SCI values by performing the Mann–Whitney *U* test for the continuous variables and the chi-squared test for the categorical variables. We obtained the DFS and OS curves using the Kaplan–Meier method. Furthermore, we performed a log-rank test to determine the survival differences between the low- and high-SCI groups. To ascertain the independent prognostic factors for DFS and OS, we executed a Cox proportional hazards analysis. The variables were evaluated using the log-rank test in a univariable analysis, and those that reached statistical significance (*p* < 0.1) were included in a multivariable analysis. The stepwise selection method was executed using the R 4.2.0 software to select the optimal subset of independent factors. We performed the aforementioned statistical analyses using the SPSS (V21.0) software platform (SPSS, Chicago, IL, USA). We also considered the statistical significance to be represented by *p* < 0.05.

Using the rms package in R V5.1–0 (Vanderbilt University, Nashville, TN, USA) [24], we constructed a predictive nomogram by incorporating the independent prognostic factors identified in the multivariable analysis for predicting the individualized OS at 3 and 5 years. To evaluate our constructed nomogram’s accuracy in terms of predicting the OS, we derived its concordance index (c-index), in addition to deriving that of the conventional TNM staging system for comparison. To visually assess the degree of consistency between the actual OS outcomes and the nomogram-derived OS predictions, calibration plots were drawn.

## 3. Results

### 3.1. Characteristics of Included Patients

Table 1 lists the included patients’ baseline characteristics. Most of the enrollees were male (*n* = 262; 91.0%), and 190 (66.0%) patients were aged <65 years. The tongue was noted to constitute the most frequent tumor location (*n* = 110; 38.2%), and the buccal mucosa was noted to constitute the second most frequent location (*n* = 96; 33.4%). Stage I, II, III, and IV OSCC was given as a diagnosis for 61 (21.2%), 39 (13.5%), 40 (13.9%), and 148 (51.4%) patients, respectively. Regarding the pathological features, PNI, ENE, LVI, and poorly differentiated (P-D) OSCC were present in 72 (25.0%), 58 (20.1%), 20 (6.9%), and 35 (12.2%) patients, respectively. Regarding adjuvant therapy, 113 (39.2%) patients underwent adjuvant chemoradiotherapy (CRT), and 39 (13.5%) patients underwent adjuvant radiotherapy (RT).

### 3.2. Analysis of ROC Curves and AUCs of Serum Indices

**Figure 1:**
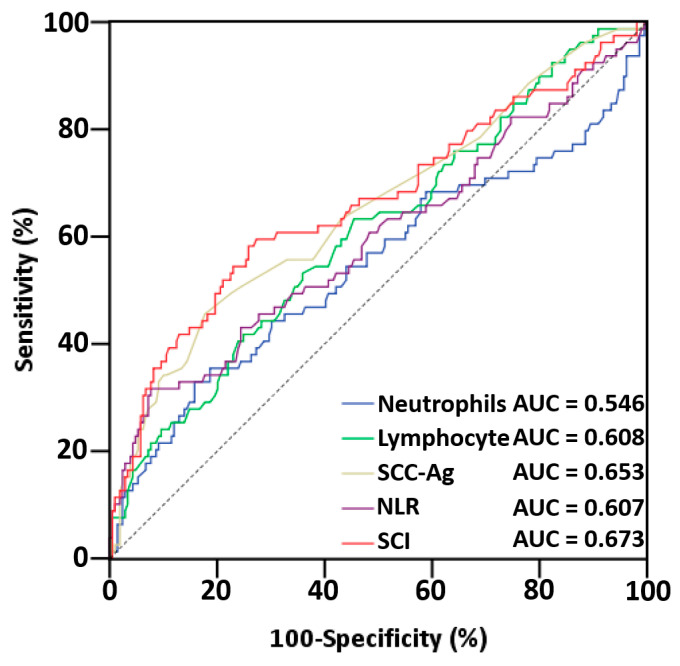
ROC curves for overall survival in patients with OSCC. Abbreviations: AUC, area under the curve; NLR, neutrophil/lymphocyte ratio; SCC-Ag, squamous cell carcinoma antigen; SCI, squamous cell carcinoma inflammatory index.

By analyzing the ROC curves, we determined the optimal SCI cutoff value for predicting the OS to be 3.45 (AUC: 0.673; 95% confidence interval [CI]: 0.593–0.749; *p* < 0.001), and the sensitivity and specificity at this value were 58.2% and 74.2%, respectively (Figure 1). Similarly, the optimal cutoff values for NLR and SCC-Ag were determined to be 4.51 (AUC: 0.607; 95% CI: 0.528–0.687; *p* = 0.005) and 1.65 (AUC: 0.653; 95% CI: 0.578–0.728; *p* < 0.001), respectively. The AUCs for the enrolled indices and their combinations, including the NLR and SCI, were also compared in terms of the predictive performance of the OS (Table 2). Most indices could predict the OS (with the exception of neutrophils; *p* = 0.228), and the AUC value derived for the SCI (0.673) was higher than the values derived for the lymphocytes (0.608), SCC-Ag (0.653), and NLR (0.607). These findings indicate that the SCI had an ideal prognostic performance level in our study setting and that the prognostic value of the SCI should be more thoroughly investigated.

### 3.3. Associations between SCI and Clinicopathological Features

The differences in the clinicopathological features between the low-SCI (<3.45) and high-SCI (≥3.45) groups are presented in Table 3. The high- and low-SCI groups had 100 (34.7%) and 188 (65.3%) patients, respectively. We noted that the high-SCI group contained a higher proportion of individuals with stage III to IV OSCC (*p* < 0.001), PNI (*p* = 0.002), ENE (*p* < 0.001), late T and N statuses (both *p* < 0.001), LVI (*p* < 0.001), the need for adjuvant therapy (*p* < 0.001), higher CCI scores (*p* = 0.029), shorter median survival (*p* < 0.001), and DOI ≥10 mm (*p* < 0.001) compared to the low-SCI group.

### 3.4. Associations between SCI and OS

**Figure 2:**
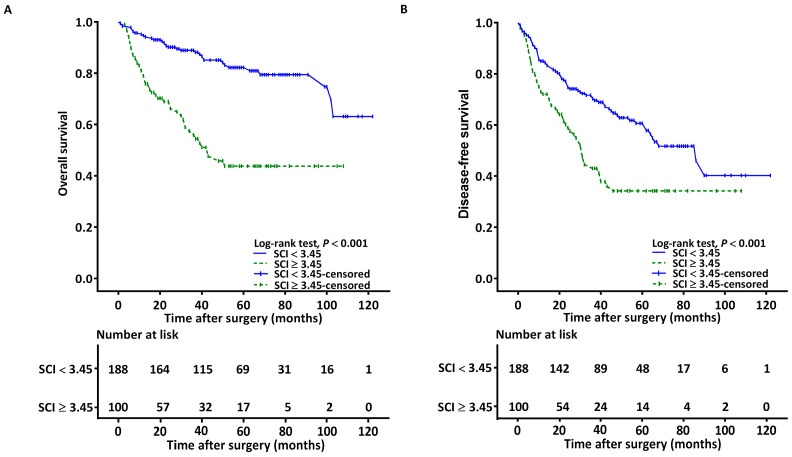
Kaplan–Meier curves for (**A**) overall survival and (**B**) disease-free survival of patients with SCI of ≥3.45 and <3.45. Abbreviations: SCI, squamous cell carcinoma inflammatory index.

The Kaplan–Meier curves revealed the estimated median OS derived for the low-SCI (<3.45) and high-SCI (≥3.45) groups to be >178 and 43.2 months (95% CI: 29.1–57.3), respectively. The log-rank test revealed significant between-group differences with regard to survival (*p* < 0.001; Figure 2A). Our univariable analysis revealed LVI, stage IV disease, the need for CRT, PNI, CCI ≥ 2, NLR ≥ 4.51, P-D, SCC-Ag ≥ 1.65, and SCI ≥ 3.45 to have a significant association with adverse OS (Table 4). Since SCC-Ag and the NLR are components of the SCI, separate multivariable analysis models were employed to prevent multicollinearity. As shown in Table 5, the results of the multivariable analysis reveal that a high SCI of ≥3.45 had an independent association with adverse OS, with the corresponding hazard ratio (HR) being 2.378 (95% CI, 1.356–3.735; *p* = 0.002). We also identified additional independent prognostic factors for adverse OS, including stage IV disease, P-D, CCI ≥ 2, SCC-Ag ≥ 1.65, and NLR ≥ 4.51.

### 3.5. Associations between SCI and DFS

The Kaplan–Meier curves revealed the estimated median DFS derived for the low-SCI (<3.45) and high-SCI (≥3.45) groups to be 86.3 months (95% CI: 64.2–108.7) and 31.2 months (95% CI: 26.1–36.3), respectively. The log-rank test revealed significant between-group differences (*p* < 0.001; Figure 2B). Table 4 presents the significant predictors of a poor DFS as identified in the univariable analysis. The predictors included the following: stage IV OSCC, NLR ≥ 4.51, P-D, the need for adjuvant CRT, SCC-Ag ≥ 1.65, LVI, and SCI ≥ 3.45. In the multivariable analysis, an SCI value of ≥3.45 was independently associated with an unfavorable DFS, with the corresponding HR (95% CI) being 2.219 (1.437–3.425; *p* < 0.001; Table 5). We also determined additional independent risk factors for an unfavorable DFS, including stage IV disease, P-D, SCC-Ag ≥ 1.65, and NLR ≥ 4.51.

### 3.6. Stratified Analysis

**Figure 3:**
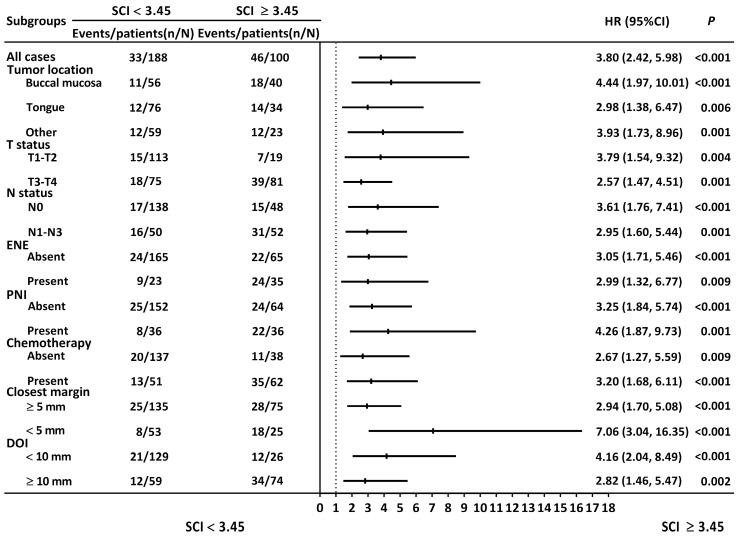
Hazard ratios for SCI in stratified analysis. Abbreviations: DOI, depth of invasion; ENE, extranodal extension; HR, hazard ratio; PNI, perineural invasion; SCI, squamous cell carcinoma inflammatory index.

We executed a stratified analysis of the SCI’s OS discriminatory ability (Figure 3). A high SCI value was consistently and significantly associated with adverse OS, regardless of whether the patient data were stratified by the tumor location (the tongue, buccal mucosa, and other locations), T status (T1 to T2 and T3 to T4; *p* = 0.004 and 0.001, respectively), N status (N0 and N1 to 3; *p* < 0.001 and 0.001, respectively), ENE status (absent or present; *p* < 0.001 and 0.009, respectively), PNI status (absent or present; *p* < 0.001 and 0.001, respectively), the need for adjuvant chemotherapy (necessary and not necessary; *p* = 0.009 and < 0.001, respectively), the closest margin (≥5 and <5 mm; both *p* < 0.001), or DOI status (<10 and ≥10 mm; *p* < 0.001 and 0.002, respectively).

### 3.7. Predictive Nomogram Construction

**Figure 4:**
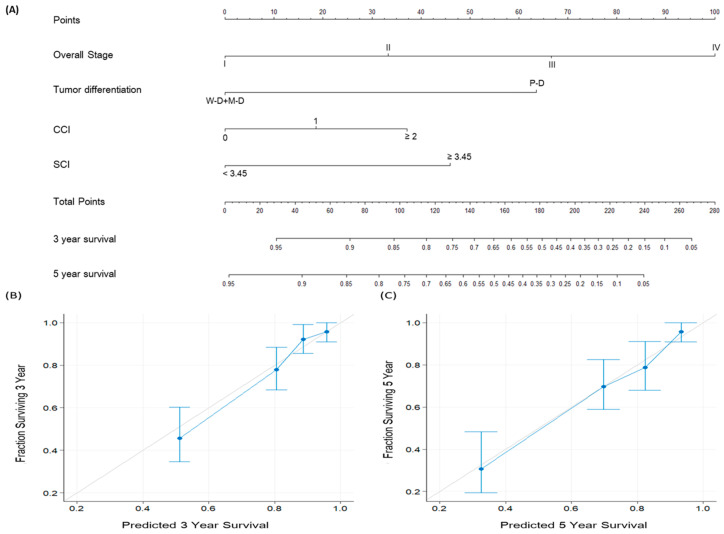
(**A**) Nomogram for OS prediction based on independent prognostic factors in multivariable analysis. The corresponding points of each variable’s line segment indicate the degree of risk contributed by this variable. The sum of points from all variables yields the total points, which can be converted to the estimated 3- and 5-year OS probabilities by drawing a vertical line from the total points to the following survival axes. The nomogram’s calibration plots for the (**B**) 3- and (**C**) 5-year OS predictions. The 45° gray line indicates the perfect OS prediction, and the blue line indicates the nomogram’s predicted outcomes. Abbreviations: CCI, Charlson comorbidity index; M-D, moderately differentiated; P-D, poorly differentiated; SCI, squamous cell carcinoma inflammatory index; W-D, well differentiated.

We constructed a nomogram by incorporating the significant prognostic factors derived from our multivariable analysis of the OS, including SCI, tumor differentiation, cancer stage, and CCI, and employed it for individualized OS predictions (Figure 4A). The SCI-based nomogram’s c-index value was 0.779 (95% CI: 0.744–0.813), whereas the c-index value for the nomogram constructed using only the TNM staging system was 0.697 (95% CI: 0.664–0.729). Calibration plots were also drawn to evaluate how close the nomogram-estimated OS was to the actual observed survival outcomes. The slopes of the predicted 3- and 5-year OS probability calibration curves (as displayed in Figure 4B,C, respectively) were noted to approximate the ideal 45° line. Accordingly, these findings verify the strong predictive performance of our constructed nomogram.

## 4. Discussion

In the present study, we introduce a novel SCI that we derived by integrating the NLR and SCC-Ag; thus, this unique cancer-related inflammatory index incorporates both host inflammatory responses and tumor-specific proteins. By enrolling 288 patients undergoing curative surgery for OSCC, we investigated the clinical role of the SCI in OSCC management and obtained several novel findings. First, the SCI had a significantly higher AUC compared to the SCC-Ag and NLR, suggesting that the SCI may be a better predictor of the OS than its individual components in OSCC prognostications. Second, our stepwise multivariable analysis demonstrated a high SCI value (≥3.45) to be an independent risk factor for unfavorable OS and DFS outcomes among individuals with OSCC. Furthermore, the SCI had consistent and significant prognostic values for OS in our stratified analyses. In summary, our hypothesis was verified, and the study results support that the novel SCI has promise as a prognostic biomarker for individuals with OSCC. Since the SCI can be easily obtained from preoperative laboratory tests, we propose that the SCI has considerable potential for use in everyday clinical practices and oncological research. Whether patients with a high SCI (≥3.45) before treatment may benefit from more personalized treatment planning warrants further investigation.

Several researchers have combined tumor markers with inflammatory indices and investigated their prognostic roles in individuals with cancer. In previous studies by our colleagues, high SCC-Ag and C-reactive protein levels can predict advanced cancer stages and adverse survival outcomes in patients with OSCC [16] and recurrent diseases [9]. Su et al. developed a novel prognostic index by multiplying carcinoembryonic antigen values and NLR values and determined that the derived index had a significant association with OS in patients undergoing regorafenib treatment for metastatic colorectal cancer [25]. Wang et al. conducted a retrospective analysis of data from 515 patients who had cervical cancer and observed that SCC-Ag had a higher predictive efficacy than the NLR for all tumor stages [26]. Our study further demonstrated that the AUC derived for the SCI was higher than that derived for the SCC-Ag and NLR. This finding suggests that the SCI has better predictive efficacy for patients with operable OSCC, presumably due to better reflecting host–tumor interactions and anti-tumor immune responses. Our study results support the idea that a comprehensive consideration of the tumor-specific factors and host inflammatory responses during prognostic evaluations can increase the accuracy of survival predictions for patients with OSCC. In the future, prospective studies should explore whether preoperative SCI values or changes in SCI values can be used to predict the prognosis of other human squamous cell cancers.

Our study findings support SCI as a unique and hopeful prognostic indicator for patients with OSCC. However, the exact mechanisms that govern the associations between an elevated SCI and adverse clinical outcomes remain unknown. An elevated SCI can be indicative of increased serum SCC-Ag levels and/or NLR values, which may provide insight into how the SCI predicts the prognosis of OSCC. Studies have reported that serum SCC-Ag exhibits an association with distant metastasis, tumor progression, and lymph node metastasis [27] and that the production of serum SCC-Ag in OSCC is attributable to lymphocytes surrounding cancer cells [28]. Lin et al. assessed 79 patients with OSCC and revealed an SCC-Ag value ≥ 2.0 ng/mL to be significantly associated with advanced tumor and nodal status and with poor OS and DFS [29]. Moreover, SCC-Ag has been reported to be able to predict the early recurrence of OSCC. In an evaluation of 100 patients’ medical records, Chen et al. demonstrated that a high SCC-Ag level could predict tumor recurrence and prognosis among individuals with recurrent OSCC [9]. This predictive effect may be explained by the involvement of SCC-Ag in tumor cell apoptosis inhibition at the molecular level [30]. Several studies have investigated the value of the NLR for predicting disease prognosis among patients who received treatment for OSCC, and this signifies that systemic inflammatory responses have an informative role in OSCC management. The mentioned studies have reported that a high NLR is associated with advanced cancer stages, a poor response to chemotherapy, and poor OS and DFS in patients with OSCC [20,31]. Furthermore, Yasumatsu et al. assessed the dynamic changes in the NLR values in 41 patients who had received nivolumab treatment for metastatic or recurrent head and neck cancer. They reported that monitoring changes in the NLR may enable the early detection of treatment failures during nivolumab monotherapies [32]. Our findings also reveal significant associations between a high SCI (≥3.45) and adverse clinicopathological features. The following mechanisms may govern the aforementioned associations: (1) a greater tumor burden (e.g., advanced tumor extension and lymph node metastasis) may be accompanied by higher cancer-related inflammation, leading to a high SCI; and (2) tumor invasion (e.g., PNI or LVI) may be promoted by SCC-Ag with the stimulation of matrix metalloprotease-9 production [33], contributing to a high SCI. The aforementioned findings provide evidence that may assist in identifying the mechanism underlying the prognostic effect of the SCI among patients with OSCC. Nevertheless, the exact mechanism warrants further exploration.

At present, the TNM staging system is the benchmark for OSCC treatment stratification and prognostication [34]. Nevertheless, the TNM staging system categorizes patients on the basis of their survival outcomes and anatomical extension, failing to account for crucial factors that govern the prognosis of OSCC, such as tumor differentiation and patients’ underlying comorbidity [35]. Given the limitations of the TNM staging system, nomograms have been proposed as an effective tool for determining disease prognoses in the era of personalized medicine [35]. Some of the main advantages of nomograms are as follows: (1) they offer pictorial presentations of clinical and pathological information with relevant weights for each variable; (2) they offer prognostic estimates that can assist in personalized treatment planning; and (3) they enable the estimation of the probability of a variety of clinical events that may affect the care of patients with cancer. The present study constructed an SCI-based nomogram that not only enables the realization of the clinical utility of the SCI but also incorporates independent prognostic factors to accurately predict individualized OSs. The SCI-based nomogram’s c-index values and calibration plots demonstrated our nomogram’s favorable performance and calibration in our OSCC cohorts. However, treatment selection for patients with OSCC should be based on clinical trials rather than nomogram data alone [35]. Accordingly, additional studies with a prospective design are warranted to verify whether the use of SCI-based nomograms for decision-making truly improves the prognosis of OSCC.

The key strength of this study is that it is the first to introduce a novel SCI that can help OSCC prognostication. Additionally, our constructed nomogram enables OS prediction that is both accurate and individualized; our nomogram also demonstrates the applicability of the SCI in clinical practice. The other strengths of the present study are its homogeneously treated cohorts and long follow-up period. However, the several limitations of our study should be acknowledged. First, the retrospective single-center study design has inherent bias. This bias can be overcome in future studies by employing national or multicenter databases. Second, we did not validate our derived study findings through the use of an independent dataset, which prevented the confirmation of the external validity of our findings. Finally, because a consensus has not been reached on the best cutoff level for the SCI, its use in clinical practice warrants further investigation. Therefore, well-designed, large, prospective, multi-institutional studies must be conducted to verify our findings and identify an ideal cutoff value for the SCI.

## 5. Conclusions

Our study results indicate that preoperative SCI is a promising biomarker with prognostic value for patients with operable OSCC, which may provide new insight into the development of cancer-related inflammatory indices. We additionally demonstrated the clinical utility of the SCI by creating an SCI-based nomogram that can provide personalized OS predictions. Future large-scale studies with prospective designs are warranted to verify our results and the utility of the SCI in predicting prognoses in other human squamous cell cancers.

## Figures and Tables

**Table 1:** Baseline characteristics of 288 patients with OSCC.

Characteristics	
Age (years)	
≥65	98 (34.0%)
<65	190 (66.0%)
Sex	
Women	26 (9.0%)
Men	262 (91.0%)
Tumor location	
Tongue	110 (38.2%)
Buccal	96 (33.4%)
Gingiva	41 (14.2%)
Retromolar trigone	15 (5.2%)
Mouth floor	11 (3.8%)
Lip	10 (3.5%)
Hard palate	5 (1.7%)
AJCC stage	
I	61 (21.2%)
II	39 (13.5%)
III	40 (13.9%)
IV	148 (51.4%)
T status	
T1	79 (27.5%)
T2	53 (18.4%)
T3	39 (13.5%)
T4	117 (40.6%)
N status	
N0	186 (64.6%)
N1	27 (9.4%)
N2	62 (21.5%)
N3	13 (4.5%)
PNI	72 (25.0%)
ENE	58 (20.1%)
LVI	20 (6.9%)
Tumor differentiation	
W-D and M-D	253 (87.8%)
P-D	35 (12.2%)
Closest margin	
≥5 mm	210 (72.9%)
<5 mm	78 (27.1%)
DOI ≥ 10 mm	133 (46.2%)
Treatment modality	
Surgery only	136 (47.3%)
Surgery + RT	39 (13.5%)
Surgery + CRT	113 (39.2%)
CCI	
0	157 (54.5%)
1	83 (28.8%)
≥2	48 (16.7%)
Personal Habits	
Smoking	241 (83.7%)
Alcohol drinking	191 (66.3%)
Areca nut chewing	233 (80.9%)
SCC-Ag (ng/mL), median (IQR)	1.00 (0.70–1.70)
Neutrophil (×10^3^/μL), median (IQR)	5.01 (3.61–6.42)
Lymphocyte (×10^3^/μL), median (IQR)	2.01 (1.60–2.59)
SCI, median (IQR)	2.45 (1.42–4.76)

Abbreviations: AJCC, American Joint Committee on Cancer; CCI, Charlson comorbidity index; CRT, chemoradiotherapy; DOI, depth of invasion; ENE, extranodal extension; IQR, interquartile range; LVI, lymphovascular invasion; M-D, moderately differentiated; OSCC, oral cavity squamous cell carcinoma; P-D, poorly differentiated; PNI, perineural invasion; RT, radiotherapy; SCC-Ag, squamous cell carcinoma antigen; SCI, squamous cell carcinoma inflammatory index; W-D, well differentiated.

**Table 2:** Comparison of the AUC values of SCI and its components.

Index	AUC	95% CI	*p*	*p* ^a^
Neutrophil	0.546	(0.464–0.628)	0.228	<0.001
Lymphocyte	0.608	(0.534–0.682)	0.005	<0.001
SCC-Ag	0.653	(0.578–0.728)	<0.001	<0.001
NLR	0.607	(0.528–0.687)	0.005	<0.001
SCI	0.673	(0.593–0.749)	<0.001	-

Abbreviations: AUC, area under the curve; CI, confidence interval; NLR, neutrophil-to-lymphocyte ratio; SCC-Ag, squamous cell carcinoma antigen; SCI, squamous cell carcinoma inflammatory index. ^a^ The AUC values between the SCI and the other factors were compared using the Z-test method. Mann–Whitney U test (Z-test: neutrophil: −6.798; lymphocyte: −6.285; SCC-Ag: −11.083; and NLR: −9.925).

**Table 3:** Clinicopathological features according to the cutoff value of SCI.

Variable	Number of Patients		
	SCI < 3.45, *n* = 188	SCI ≥ 3.45, *n* =100	*p*
Sex			0.191 ^a^
Women	20 (10.6%)	6 (6.0%)	
Men	168 (89.4%)	94 (94.0%)	
Age			0.194 ^a^
<65	129 (68.6%)	61 (61.0%)	
≥65	59 (31.4%)	39 (39.0%)	
AJCC stage			<0.001 ^a^
I–II	91 (48.4%)	9 (9.0%)	
III–IV	97 (51.6%)	91 (91.0%)	
T status			<0.001 ^a^
T1–T2	113 (60.1%)	19 (19.0%)	
T3–T4	75 (39.9%)	81 (81.0%)	
N status			
N0	138 (73.4%)	48 (48.0%)	<0.001 ^a^
N1–N3	50 (26.6%)	52 (52.0%)	
PNI			
Absent	152 (80.9%)	64 (64.0%)	0.002 ^a^
Present	36 (19.1%)	36 (36.0%)	
LVI			
Absent	183 (97.3%)	85 (85.0%)	<0.001 ^a^
Present	5 (2.7%)	15 (15.0%)	
ENE			<0.001 ^a^
Absent	165 (87.8%)	65 (65.0%)	
Present	23 (12.2%)	35 (35.0%)	
Tumor differentiation			0.145 ^a^
W-D/M-D	169 (89.9%)	84 (84.0%)	
P-D	19 (10.1%)	16 (16.0%)	
Closest margin			0.562 ^a^
≥5 mm	135 (71.8%)	75 (75.0%)	
<5 mm	53 (28.2%)	25 (25.0%)	
DOI ≥ 10 mm			<0.001 ^a^
No	129 (68.6%)	26 (26.0%)	
Yes	59 (31.4%)	74 (74.0%)	
Tumor location			0.215 ^a^
Tongue	76 (40.4%)	34 (34.0%)	
Buccal mucosa	56 (29.8%)	40 (40.0%)	
Other	56 (29.8%)	26 (26.0%)	
Personal habits			0.808 ^a^
No exposure	20 (10.6%)	13 (13.0%)	
One exposure	10 (5.3%)	6 (6.0%)	
Two or all exposure	158 (84.0%)	81 (81.0%)	
Treatment modality			<0.001 ^a^
Surgery	111 (59.0%)	25 (25.0%)	
Surgery + RT	26 (13.8%)	13 (13.0%)	
Surgery + CRT	51 (27.1%)	62 (62.0%)	
CCI			0.029 ^a^
0	111 (59.0%)	46 (46.0%)	
1	53 (28.2%)	30 (30.0%)	
≥2	24 (12.8%)	24 (24.0%)	
Survival in months, median (IQR)	49.00 (30.25–70.75)	25.00 (13.00–48.00)	<0.001 ^b^

Abbreviations: AJCC, American Joint Committee on Cancer; CCI, Charlson comorbidity index; CRT, concurrent chemoradiotherapy; DOI, depth of invasion; ENE, extranodal extension; IQR, interquartile range; LVI, lymphovascular invasion; M-D, moderately differentiated squamous cell carcinoma; PNI, perineural invasion; P-D, poorly differentiated squamous cell carcinoma; RT, radiotherapy; SCI, squamous cell carcinoma inflammatory index; W-D, well differentiated squamous cell carcinoma. ^a^ chi-square test. ^b^ Mann–Whitney U test.

**Table 4:** Univariable analysis of the prognostic factors for OS and DFS.

Variable	Univariable Analysis (OS)		Univariable Analysis (DFS)	
	HR (95% CI)	*p*	HR (95% CI)	*p*
Sex				
Women	Reference		Reference	
Men	1.584 (0.640–3.921)	0.320	1.316 (0.640–3.921)	0.320
Age (years)				
<65	Reference		Reference	
≥65	0.760 (0.468–1.236)	0.269	0.684 (0.465–1.006)	0.053
AJCC stage				
I	Reference		Reference	
II	1.287 (0.392–4.219)	0.677	0.649 (0.305–1.379)	0.261
III	1.010 (0.284–3.585)	0.988	0.938 (0.469–1.876)	0.857
IV	5.831 (2.250–13.493)	<0.001	2.120 (1.313–3.422)	0.002
Presence of PNI				
No	Reference		Reference	
Yes	2.294 (1.453–3.622)	<0.001	1.336 (0.905–1.973)	0.144
Presence of LVI				
No	Reference		Reference	
Yes	3.707 (1.943–7.071)	<0.001	1.882 (1.010–3.505)	0.046
Tumor differentiation				
W-D/M-D	Reference		Reference	
P-D	3.260 (1.935–5.492)	<0.001	2.224 (1.410–3.508)	0.001
Treatment modality				
Surgery	Reference		Reference	
Surgery + RT	1.400 (0.625–3.135)	0.414	0.854 (0.466–1.565)	0.609
Surgery + CRT	3.300 (2.004–5.434)	<0.001	1.633 (1.131–2.357)	0.009
Tumor location				
Tongue	Reference		Reference	
Buccal mucosa	1.319 (0.776–2.240)	0.306	0.854 (0.466–1.565)	0.609
Other sites	1.171 (0.672–2.040)	0.577	1.633 (0.531–2.357)	0.439
Closest margin				
≥ 5 mm	Reference		Reference	
< 5 mm	1.325 (0.826–2.124)	0.243	1.278 (0.881–1.854)	0.196
Personal habits				
No exposure	Reference		Reference	
One exposure	2.213 (0.713–6.867)	0.169	2.220 (0.855–5.764)	0.101
Two or more exposure	1.540 (0.668–3.551)	0.311	1.919 (0.972–3.791)	0.060
CCI				
0	Reference		Reference	
1	1.057 (0.615–1.817)	0.841	0.682 (0.441–1.053)	0.084
≥2	1.916 (1.121–3.273)	0.017	1.058 (0.672–1.667)	0.808
SCC-Ag				
<1.65	Reference		Reference	
≥1.65	3.521 (2.250–5.512)	<0.001	2.006 (1.390–2.893)	<0.001
SCI				
<3.45	Reference		Reference	
≥3.45	3.803 (2.419–5.980)	<0.001	1.900 (1.335–2.703)	<0.001
NLR				
<4.51	Reference		Reference	
≥4.51	4.465 (2.762–7.219)	<0.001	2.710 (1.783–4.118)	<0.001

Abbreviations: AJCC, American Joint Committee on Cancer; CCI, Charlson comorbidity index; CI, confidence interval; CRT, chemoradiotherapy; DFS, disease-free survival; HR, hazard ratio; LVI, lymphovascular invasion; M-D, moderately differentiated; NPAR, neutrophil percentage-to-albumin ratio; OS, overall survival; P-D, poorly differentiated; PNI, perineural invasion; RT, radiotherapy; SCI, squamous cell carcinoma inflammatory index; W-D, well differentiated.

**Table 5:** Multivariable analysis of the prognostic factors for OS and DFS.

Variable	SCC-Ag and NLR Model		SCI Model	
	HR (95% CI)	*p*	HR (95% CI)	*p*
Overall Survival				
AJCC stage				
I	Reference		Reference	
II				
III				
IV	3.702 (2.028–6.758)	<0.001	3.935 (2.148–7.209)	<0.001
Tumor differentiation				
W-D/M-D	Reference		Reference	
P-D	2.976 (1.738–5.095)	<0.001	2.855 (1.677–4.861)	<0.001
CCI				
0	Reference		Reference	
1				
≥2	1.265 (1.147–1.689)	0.021	1.309 (1.183–1.744)	0.016
SCC-Ag				
<1.65	Reference			
≥1.65	1.905 (1.164–3.118)	0.011		
SCI				
<3.45			Reference	
≥3.45			2.378 (1.356–3.735)	0.002
NLR				
<4.51	Reference			
≥4.51	2.611 (1.580–4.315)	<0.001		
Disease-free survival				
AJCC stage				
I	Reference		Reference	
II				
III				
IV	2.326 (1.436–3.768)	<0.001	1.961 (1.320–2.913)	<0.001
Tumor differentiation				
W-D/M-D	Reference		Reference	
P-D	2.238 (1.403–3.570)	<0.001	2.061 (1.302–3.262)	0.002
SCC-Ag				
<1.65	Reference			
≥1.65	1.546 (1.041–2.295)	0.031		
SCI				
<3.45			Reference	
≥3.45			2.219 (1.437–3.425)	<0.001
NLR				
<4.51	Reference			
≥4.51	2.051 (1.361–2.867)	0.003		

Abbreviations: AJCC, American Joint Committee on Cancer; CCI, Charlson comorbidity index; CI, confidence interval; HR, hazard ratio; M-D, moderately differentiated; NLR, neutrophil-to-lymphocyte ratio; P-D, poorly differentiated; SCC-Ag, squamous cell carcinoma antigen; SCI, squamous cell carcinoma inflammatory index; W-D, well differentiated.

## Data Availability

The data presented in this study are available from the corresponding author upon request.
